# Pandemien — was kann man (ökonomisch) in Zukunft besser machen?

**DOI:** 10.1007/s10273-021-2974-6

**Published:** 2021-08-24

**Authors:** ZG Überblick

**Keywords:** I10, I18, H84

## Abstract

Die COVID-19-Pandemie hat die Fragilität des Gesundheitssystems, der Gesellschaft und der Wirtschaft beim Auftreten von weltweiten Krisen deutlich gemacht. Um auf die nächste Krise besser vorbereitet zu sein, stellen sich gesundheitspolitische, wirtschaftliche, rechtliche, ethische und politische Fragen. Zu diesem Thema veranstaltete die Berlin-Brandenburgische Akademie der Wissenschaften (BBAW) am 29. Juni 2021 ein Symposium. Gibt es bessere Strategien für mehr Resilienz, Effektivität und Gerechtigkeit? Was kann man dem Markt überlassen, was ist Sache des Staates? Wie sortiert man Finanzierung, Verantwortlichkeiten und Entscheidungsrechte innerhalb eines Landes und über die (europäischen) Ländergrenzen hinweg. Ausgewählte Beiträge zum Symposium werden in diesem Wirtschaftsdienst-Zeitgespräch zusammengefasst.

## Abstract (en)

The COVID-19 pandemic has highlighted the fragility of the health system, society and economy when global crises occur. In order to be better prepared for the next crisis, health policy, economic, legal, ethical and political questions must be answered. The Berlin-Brandenburg Academy of Sciences and Humanities (BBAW) organised a symposium on this topic on 29 June 2021. Are there better strategies for more resilience, effectiveness and justice? What can be left to the market, and what is a matter for the state? How should funding, responsibilities and decisionmaking rights be sorted within a country and across (European) national borders? Selected contributions to the symposium are summarised in this Wirtschaftsdienst-Zeitgespräch.

